# A flavor uniformity evaluation and improvement of Chinese spirit by electronic nose

**DOI:** 10.1002/fsn3.1436

**Published:** 2020-02-11

**Authors:** Jing Li, Zhenfeng Li, Xia Deng, Chunfang Song, Vijaya Raghavan, Yufei Xie

**Affiliations:** ^1^ Jiangsu Key Laboratory of Advanced Food Manufacturing Equipment & Technology School of Mechanical Engineering Jiangnan University Wuxi China; ^2^ Beijing Advanced Innovation Center for Food Nutrition and Human Health Beijing Technology and Business University (BTBU) Beijing China; ^3^ Department of Biosource Engineering McGill University Sainte‐Anne‐de‐Bellevue QC Canada

**Keywords:** automatic blending, Chinese spirits, electronic nose, flavor chemistry

## Abstract

Fenjiu spirits are famous for their multifarious flavors in China. Nevertheless, the uniformity and homogeneity of the products are always challenges for the producers. Three flavor measurement methods, that is, zNose™, GC‐MS, and spirit tasters, were employed and correlated for evaluation of Fenjiu flavors' difference. An automatic blending system was designed to improve the uniformity, where base liquors of Dazongjiu, Dajiu, and Daijiu, as well as pure water, were used for blending, and BPNN was employed for regression and error minimization. Results showed that zNose™ results could be correlated with GC‐MS in 95%; hence, zNose™ can replace GC‐MS in Fenjiu flavor measurement. With the zNose™ assistance, an experimental scale, fully automatic blending system could be optimized with different modern mathematical algorithms to improve the Fenjiu products uniformity. We believe that this detailed work will advance the scientific knowledge in the field and help to facilitate the uniformity and homogeneity of Fenjiu products.

## Graphical abstract

Three flavor measurement methods, that is, GC‐MS, liquor tasters, and zNose, were employed for evaluation of flavors difference between the same Chinese spirit products. zNose was correlated with GC‐MS in 95% and could be a replacement of GC‐MS in Fenjiu flavor measurement. An automatic blending system with the zNose assistance was designed to improve the Fenjiu products uniformity.

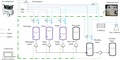

## 1 INTRODUCTION

Chinese spirits are famous for their multifarious flavors, which differentiate themselves from other liquors in the world. Up to date, 1,874 kinds of volatile flavor compounds have been identified in Chinese spirits, and the number is still increasing with new advanced analytical techniques (Fan & Qian, 2006; liu & Sun, 2018; Zhao, Liu, Shu, & He, 2019). Specialists attempt to classify Chinese spirits into different types by corresponding flavors characteristics, while disputes always exist (Liu, 1992; Wu, 2001; Yu, 2015). Among them, “Fenjiu” is the representative of light flavor style and has a history of over 1,000 years (Bai, 2017; Guo & Wang, 2006). Currently, Fenjiu has nine series and 113 products, which are graded by their flavors and quality levels (Fenjiu Group, 2018).

Fenjiu is produced in a special process of twice solid fermentation and twice distillation, which makes its flavor distinctive (Ren & Qu, 2014; Wu, Zheng, Han, Vervoort, & Nout, 2009). The major production process includes the following steps with repetition: ingredient formulation; grinding and cooking; mixing and cooling; mixing with Daqu; loading to the fermentation vessel; alcoholic fermentation; distillation; and aging (Zheng & Han, 2016). Finally, various base liquors with flavoring liquor and pure water are blended to formulate the final Fenjiu products. Nevertheless, as an agricultural produce, the uniformity and homogeneity of Fenjiu products are always a challenge for the producers. This means, the named same product may have different flavor and quality (Jiang, Peng, & Peng, 2010; Yu, Sun, Wang, & Huang, 2018; Zhang, Rao, & Li, 2016).

The major causes may include the following:

1. The production season: Fenjiu is produced in three seasons of autumn, winter, and spring. Generally, products of different seasons possess obvious different flavors and quality;
2. The base liquors' production process: In the above production steps, each step may induce difference due to the diversity and complexity of raw materials. In addition, workers' experience, equipment, and aging condition are the potential treats cause the flavor differences of the base liquors;
3. The spirit tasters: In classifying, grading, and blending of the final Fenjiu products, spirit tasters play the key role. However, as human beings, they are individually influenced by age, health status, emotion, fatigue, and even weather condition, which may lead to inaccurate judgment (Deng, Li, Wang, Song, & Zhang, 2017; Li, Ge, & Sun, 2017). All these situations occur in the entire Chinese spirits industry, which makes the spirits flavors and quality unstable and unreliable. With the novel technologies development, it is a good time now to find a solution to improve the uniformity of Chinese spirits to benefit this old industry.

Blending is the last but most important step in spirits' production. A sound blending should compensate the imperfection of the base liquors and improve the flavor and quality uniformity. The cost is also considered in this step where low‐price base liquors should be used in priority, but good flavors and quality should be retained. However, the traditional “trial to scale” method is still widely adopted in spirits industry, and the evaluation criterion mainly depends on the spirit tasters (Huang, Sun, & Su, 2014). This method has shown its weakness in quality assurance due to the above reasons. Moreover, a good cost efficiency is hard to achieve because of the complexity and large variations of the base liquors (Peng & Pan, 2015; Yuan, Zhao, Chen, Chen, & Li, 2018). Some modern blending methods have been attempted with assistance of GC‐MS and computation algorithms, that is, fuzzy logic, neural network, and objective programming (Xie & Li, 2007; Yang, Wu, Huang, & Huang, 2010; Zhang, Meng, & Li, 2011). However, fast and online measurement of flavors in blending process has not been reported in literature, and a fully automatic blending operation has not been implemented up to date. Also, those methods reported in literature are confined to unaltered base liquors and cannot adapt to varying raw materials. Furthermore, the blending effects cannot be verified online, and error cannot be modified instantly.

This study is aimed to evaluate the flavors difference and attempt a solution to improve the uniformity. Various tools will be employed, compared, and correlated for Fenjiu flavors measurements. A zNose™ is introduced for fast, nondestructive, and online flavors detection, and its accuracy will be testified. With the zNose™ assistance, an experimental scale, fully automatic blending system was designed and optimized with different modern mathematical algorithms to improve the Fenjiu products uniformity.

## 2 MATERIALS AND METHODS

### 2.1 Fenjiu samples

Five hundred and seventy‐one samples were used (Table 1), including 90 finish products of different grades, 235 finish products of different styles, 227 base liquors, and 19 auto‐blended products.

**Table 1:** (a) Summary of all Fenjiu samples. (b) Sensory evaluation scores of different grades' Fenjiu samples. (c) Different months' and different styles' Fenjiu samples. (d) Different batches of the base liquors

(a)				
No	Character		Total	Sources
1	The finish products of different grades		90	Shanxi Xinghuacun Fenjiu Group Limited Liability, Shanxi, China
2	The finish products of different styles	A‐Style	95	
		B‐Style	80	
		C‐Style	60	
3	Base liquor	Dazongjiu	75	
		Dajiu	76	
		Daijiu	76	
4	Auto‐blended samples		19	Automatic blending system

(b)								
No. 1	Sensory scores	60‐65	66‐70	71‐75	76‐80	81‐85	86‐90	90‐95
	Quantity	8	8	12	9	9	17	27

(c)							
	Style	January	March	June	October	December	Total
No. 2	A	/	25	25	25	20	95
	B	/	20	25	20	20	85
	C	10	15	10	10	15	60

(d)	Type	1st batch	2nd batch	3rd batch	Total
No. 3	Dazongjiu^a^	25	25	25	75
	Dajiu^b^	25	25	26	76
	Daijiu^c^	25	25	26	76

a Dazongjiu: the major aged base liquor.

b Dajiu: low‐quality‐aged liquor for cost consideration.

c Daijiu: high‐quality‐aged liquor.

Ninety Fenjiu samples of different grades were randomly selected, and their sensory scores were given by ten expert spirit tasters in Fenjiu Group according to their enterprise standard (Table 1b). To demonstrate the flavor difference among the same grade products, three styles products (A, B, and C) produced in the same year but in difference months were selected (Table 1c). Three different batches of the base liquors were obtained for their difference evaluation (Table 1d). These base liquors will also be used in automatic blending experiments. Nineteen samples were taken from finished liquor tank of automatic blending system.

All samples were supplied by Shanxi Xinghuacun Fenjiu Group Limited Liability. Mean value of three measurements was used for analysis.

### 2.2 Flavor measurements

Ten state‐level Chinese spirit tasters were selected from the tasters' team in Fenjiu Group to form a panelist, with five males and five females. Sensory scores of 1–100 in six aspects were graded according to Fenjiu enterprise standard which is listed in Table 2. The final grades were the average of ten results.

**Table 2:** Sensory score standards of Fenjiu Group

Sensory	Score	Max
Color		
Clear and transparent	5	5
Turbid	3	
Deposited solids	2	
Suspended solids	1	
Flavor		
Typical	20	20
Light and much pure	19	
Light and pure	18	
Light and less pure	17	
Light but not pure	15	
Light but exotic	14	
Less light and less fragrance	13	
Unpleasant	12	
Taste		
Base taste		
Taste fine	20	20
Less enduring	18	
Less sweet	18	
Thin and tasteless	17	
After taste		
Fine	20	20
Short	18	
Thin	18	
Bitter	17	
Odor		
Odorless	20	20
Puckery	15	
Caramel	17	
Auxiliary	15	
Fusel oil	15	
Bran	15	
Other	14	
Style		
Typical	5	5
Obvious	4.5	
Fair	4	
Stagger	2	
Body		
Full and perfect	5	5
Less full but perfect	4.5	
Less perfect	4	
Less full	3.5	
Individuality		
Obvious and pleasant	5	5
Less obvious but acceptable	4.5	
Not obvious	4	
Not acceptable	3	

An ISQ GC‐MS (Trace1310‐ISQ LT, Thermo Fisher Scientific) was used to identify the flavor chemicals in Fenjiu finished samples, as well as in the base liquors. The parameters were set as follows: The column temperature was 40°C at the beginning, raised to 250°C at a rate of 40°C/min, then raised to 300°C at a rate of 6.5°C/min, and held for 18 min. The flow rate of carrying helium gas was 1.3 ml/min at the beginning, raised to 2 ml/min at a speed of 1 ml/min, and then kept for 15 min. The PTV sampling temperature was 40°C at the beginning, raised to 320°C at a rate of 14.5°C/min, held for 27 min, then raised to 350°C at a rate of 14.5°C/min, and held for 3 min. An EI ion source was adapted, and its temperature was set to 230°C.

A zNose™ (Electronic Sensor Technology) was used for fast flavor detection, which can detect a flavor in 1 min and send the detected signals to a PC instantly. The signals were illustrated in the form of multipeaks, which was similar to a GC, and could also form a fingerprint for a specific flavor. Its operation parameters were set as follows: senor detection temperature at 60°C, sensor baking temperature 150°C, and column temperature was raised from 40 to 180°C at a rate of 10°C/min. The flow rate of helium gas was 0.03 ml/s. Details of zNose™ can be found in literatures (Gan, Man, Tan, NorAini, & Nazimah, 2005; Li, Wang, Raghavan, & Vigneault, 2011).

### 2.3 Mathematical methods

Pearson correlation coefficient (SPSS 19.0, IBM) was analyzed for the peak areas of GC‐MS and zNose™. A *t* test was conducted with *r* and *p* values reported; multilinear line regression (MLR) was employed to model the sensory scores with zNose™ peaks; analysis of means (ANOM) was applied to base liquors where means (MN) and coefficient variable (CV) were used to select the representative peaks for automatic blending.

Principle component analysis (PCA) of zNose™ peaks was conducted for differentiation of the Fenjiu samples. The new PCs were analyzed and displayed in three‐dimensional scores plots in this study. The main purpose of PCA is dimension reduction to obtain a few new variables that can represent the original variables without loss of information as much as possible (Ezhilan, Nesakumar, & Babu, 2018; Ickes & Cadwallader., 2018; Jiang, Wang, Wang, & Cheng, 2017; Verma & Panda, 2018; Zhang et al., 2017). Further, analysis of means (ANOM) was conducted for significant difference from the overall mean. Mean value (MN), standard deviation (*SD*), and variable coefficient (CV) were used as the criteria (Equation 1), where CV <0.15 was considered as a small variation, 0.16–0.35 as medium, and larger than 0.36 as strong variation (Brown, 2011). Large CV reflected a large difference among different base liquors.

(1) $$\text{CV}=\frac{\sqrt{\frac{1}{N}\sum_{i=1}^{N}\left(x_{i}-\bar{x}\right)}}{\bar{x}}=\frac{\mathrm{SD}}{\text{MN}}\times 100\%$$

*x*: represented the peak areas, *N*: the total peak numbers.

A three‐layered feed‐forward back propagation neural network (BPNN) algorithm (MATLAB, 7.0) was constructed with an architecture of 3 × 5 × 3, by *trial and error*. It was first used to build mathematical models between spirit tasters and zNose™ and then to adjust the proportions of base liquors in automatic blending operation. BPNN is a supervised learning algorithm which needs training and validation. Weights of neurons are adjusted to minimize the mean square error between the predicted and desired output values. This method is considered to be best suited for agricultural products where nonlinear relationship and multivariable problems without regularity need to be addressed.

### 2.4 Automatic blending system

**Figure 1:**
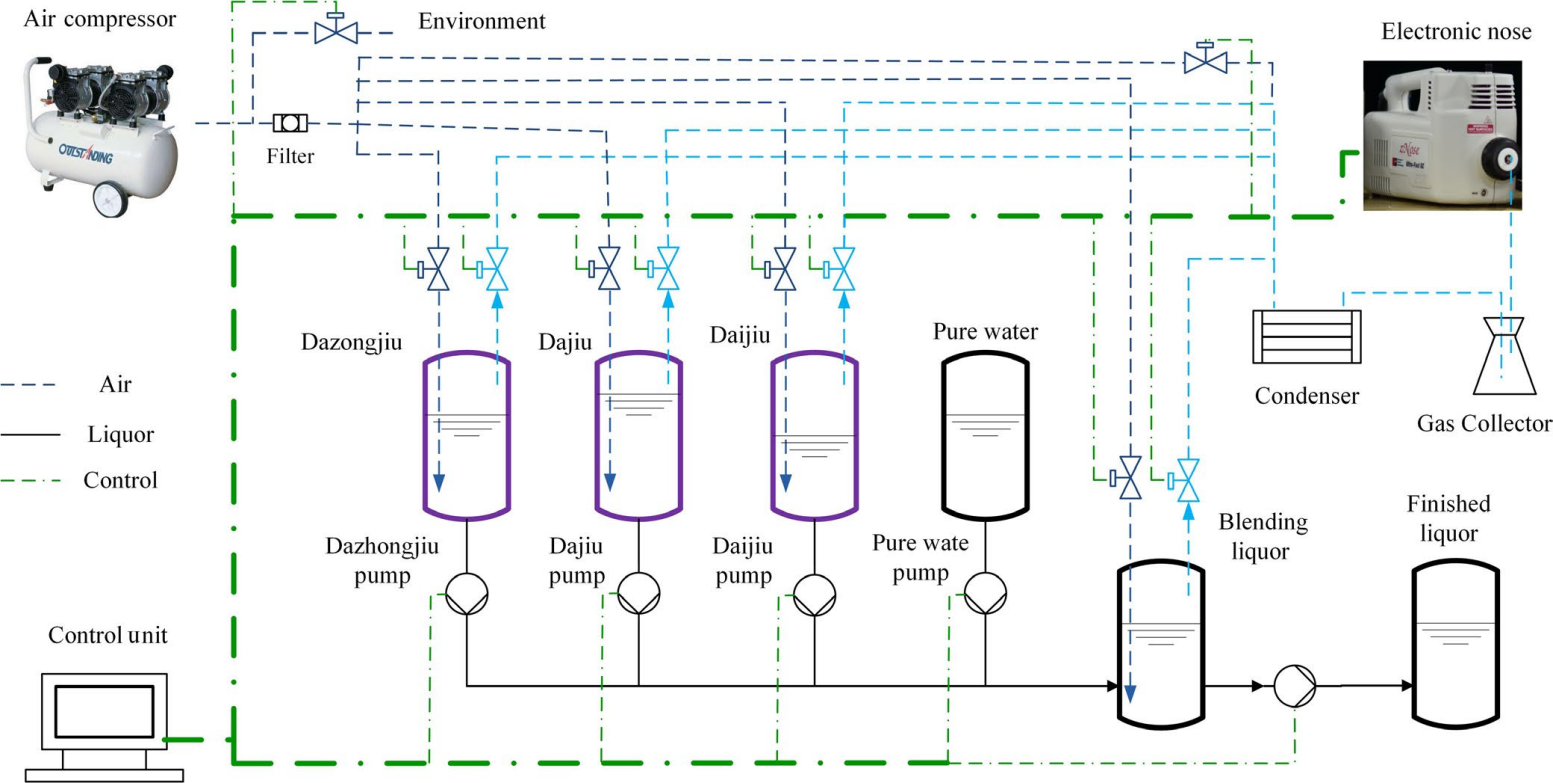
Schematic diagram of automatic blending system

To improve the uniformity of Fenjiu products, an automatic blending system was designed. Its schematic diagram is shown in Figure 1.

The system included four major parts: (a) liquor flowing unit: Base liquors (Dazongjiu, Dajiu, and Daijiu) and pure water were put in four jars separately, and they were pumped into the blending tank through low‐rate‐controlled pumps and finally collected in the finished Fenjiu tank. All liquid levels were monitored with liquid pressure sensors mounted in the bottom to ensure that enough liquids were left in jars for blending accuracy; (b) flavor detection unit: zNose™ detected the flavors of three base liquors and the blending liquor in turn. Flavor data were transferred to the control unit for signal analysis and for feedback control of the liquor flow rate; in addition, flavors were brought to zNose™ by compressed air. Gas solenoid valves were controlled in sequence to transfer each flavor to zNose™ in turn; (c) control unit: LabVIEW data acquisition card (NISB6001, NI) and software were used for the control unit. MATLAB was employed for BPNN calculation, and the results were transferred to LabVIEW for process control. The details of the system can be found in our previous study (Deng, 2018).

**Figure 2:**
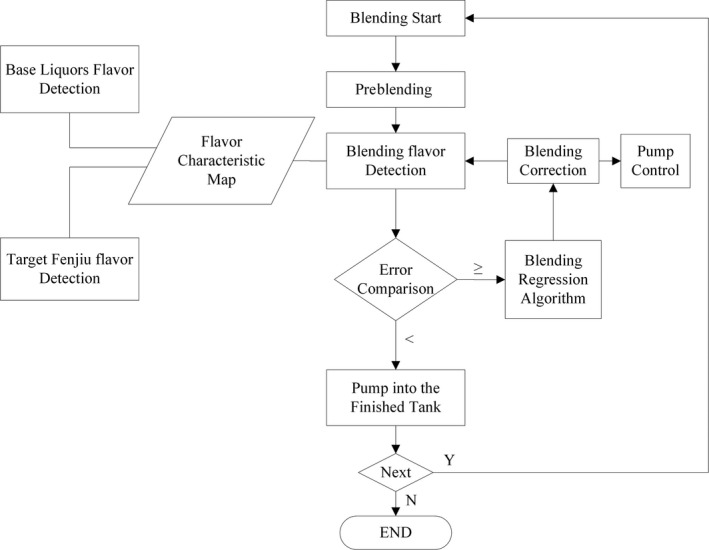
Flavor control flow chart of automatic blending system

A Fenjiu B‐style product was selected as the “Target Fenjiu” in this study. Its flavor was detected with zNose™, and the flavor fingerprint was stored in the flavor characteristic map. In blending process, all base liquors and blending Fenjiu flavors were detected in turn. The control objective was to approach the target fingerprint flavor through online adjustment of the base liquors' flow rates. When the target flavor was approached, the blending process finished. The blending flow chart is shown in Figure 2. Three common peaks of target Fenjiu and base liquors detected with zNose™ were selected for BPNN algorithm.

## 3 RESULTS AND DISCUSSION

### 3.1 Correlation analysis of GC‐MS, spirit tasters, and zNose™

The correlation of three flavor measurement methods (GC‐MS, spirit tasters, and zNose™) would be analyzed through the different grades' 90 Fenjiu samples (Table 1). Different regression models would be built for the purpose of mutual replacement of each other, as only zNose™ could be used for fast flavor detection in automatic blending system.

#### 3.1.1 GC‐MS versus zNose™

A standardized *n*‐alkanes of C6‐C14 were employed to calibrate zNose™. The evaporation points of C6‐C14 were used as references to group the Fenjiu flavor substances measured with GC‐MS. Flavor substances identification by zNose™ and GC‐MS was discussed, and Kovat's index was used to build the relationship between zNose™ and GC‐MS (Chen, Song, Bi, Meng, & Wu, 2018; Cui, Wang, Yang, Wu, & Wang, 2015; Dong et al., 2019).

**Figure 3:**
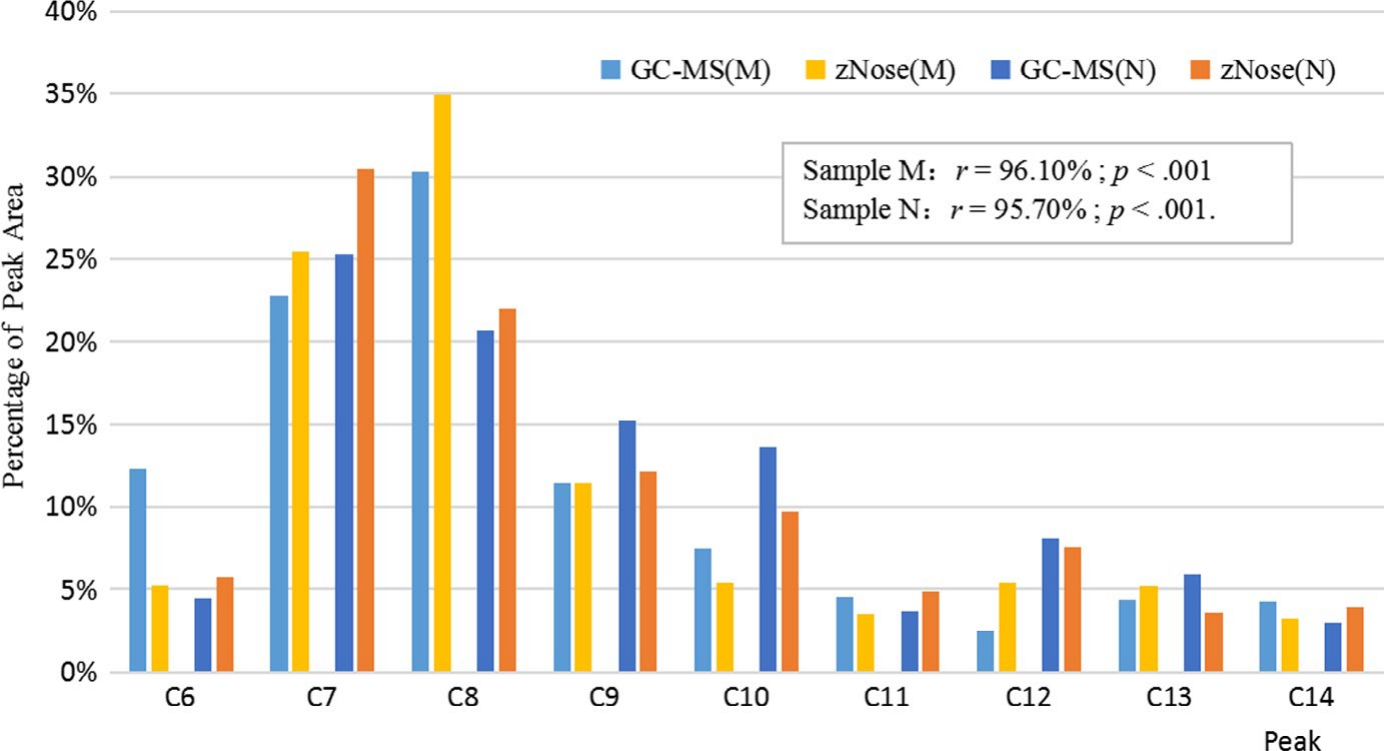
Percentage of peak area of GC‐MS and zNose™

Ninety flavor substances were identified by GC‐MS and grouped into C6‐C14 according to their evaporation points (Table 3). The substances' peak areas in each group were accumulated, and their percentages versus total peak areas were calculated and listed. Nine peaks of C6‐C14 were also detected with zNose™, and their areas' percentages versus their total areas were also listed in Figure 3 for comparison (two samples, named M and N, respectively, were selected as the representatives of the 90 Fenjiu samples). Pearson correlation coefficient *r* was calculated, as well as *p* (significant) values (Asuero, Sayago, & González, 2006). For Fenjiu sample M, *r* = 96.10% and *p* < .001; for N, *r* = 95.70%, and *p* < .001. This shows that for Fenjiu samples, M and N, zNose™, and GC‐MS measurement results are highly correlated and can replace each other in high accuracy. The same experiments were conducted for all other samples with similar results, but the report is neglected.

**Table 3:** Classification of the flavor substances measured with GC‐MS

zNose™ detected peaks	GC‐MS measured substances
C6	Acetaldehyde, Isobutyraldehyde, Triethyl orthobutyrate, Ethyl 3‐hexenoate, Ethylidenediacetate, 2‐methylbutylhexanoate, 2‐methyl‐2‐butyrobutyl ester,
C7	2‐methylpropanol, Ethyl propionate, Ethyl 2‐methylpropionate, Furfural, Diethyl succinate, 2‐methylbutyraldehyde, Isovaleraldehyde, Valeraldehyde, valeric acid, acetal, isobutanol, dihexyl diacetate, ethyl 3‐hydroxyhexanoate, ethyl undecanoate, propanol, 2‐dimethylbutyric acid, Ethyl isobutyrate, ethyl acetate
C8	1‐pentanol, ethyl butyrate, ethyl 2‐methylbutyrate, acetic acid, hexanal, ethyl butyrate, isobutyl acetate, butyl acetate, ethyl butyrate, ethyl isobutyrate, oxime Ethyl acetate, ethyl isovalerate, 2‐methylbutyrate, isoamyl alcohol, acetic acid
C9	1‐hexanol, ethyl valerate, propionic acid, 2‐methylpropionic acid, isoamyl acetate, amyl acetate, ethyl valerate, heptaldehyde, butyl isobutyrate, n‐hexanol, 3‐methyl 1‐pentanol, 3‐methyl butanol
C10	Ethyl lactate, 1‐heptanol, ethyl caproate, hexyl acetate, butyric acid, benzaldehyde, octanal, Butyl lactate, isoamyl butyrate
C11	1‐octanol, ethyl heptanoate, phenylacetaldehyde, ethyl heptanoate, 2‐ethylbutyric acid, lactic acid, isoamyl ester
C12	Ethyl caprylate, 1‐decanol, isoamyl hexanoate, octyl acetate, ethyl benzoate, naphthalene, Benzyl alcohol, Acetyl acetate, Furfural, Diethyl succinate, β‐Phenylethanol
C13	Ethyl decanoate, ethyl decanoate, ethyl phenylacetate, 1‐decanol, pentyl hexanoate, 2‐phenylethyl acetate, 4‐ethylguaiacol
C14	2‐methylpropyl acetate, ethyl cinnamate, ethyl phthalate, amyl octanoate

#### 3.1.2 Spirit tasters versus zNose™

The detected peak areas by zNose™ were also correlated with the sensory scores. MLR and BPNN were employed to build regression models, respectively. The 90 Fenjiu samples were separated into two groups: The first group included 60 samples which were randomly selected as training data, while the other 30 samples as validating data.

In MLR, nine peaks detected with zNose™ were all included. Peak areas were used as *X_i_* and the corresponding sensory scores as *Y*. A *R* ^2^ of .719 and *SD* of 4.22 were obtained which showed a low correlation between them. The respective *t* and *p* values of the MLR model are shown in Table 4. It can be observed that four peaks of C7, C9, C12, and C13 have the *p* values of <.05, indicated that they can be used as the characteristic peaks of Fenjiu samples. Hence, they were selected and again conducted in a new MLR regression model. Then, four peaks were included in MLR and the results are shown in Table 4, where *t* was increased, and *p* was decreased greatly. A *R* ^2^ of .813 and *SD* of 4.78 were obtained which showed a higher accuracy. When the 30 validating data were tested, a *R* ^2^ of .78 and *SD* of 4.72 were achieved which demonstrated an acceptable prediction.

**Table 4:** Coefficient of MLR regression models^a^ with 9 peaks and 4 peaks

Peaks	Variables	*t*	*p*
9 peaks	Constant	4.565	.000
	C6	−0.099	.921
	C7	4.716	.000
	C8	−0.889	.378
	C9	5.147	.000
	C10	1.605	.114
	C11	1.070	.289
	C12	−0.589	.043
	C13	3.437	.001
	C14	2.328	.558
4 peaks	Constant	4.219	.000
	C7	9.292	.000
	C9	3.446	.001
	C12	5.629	.000
	C13	5.147	.001

a Dependent variable: sensory scores.

**Figure 4:**
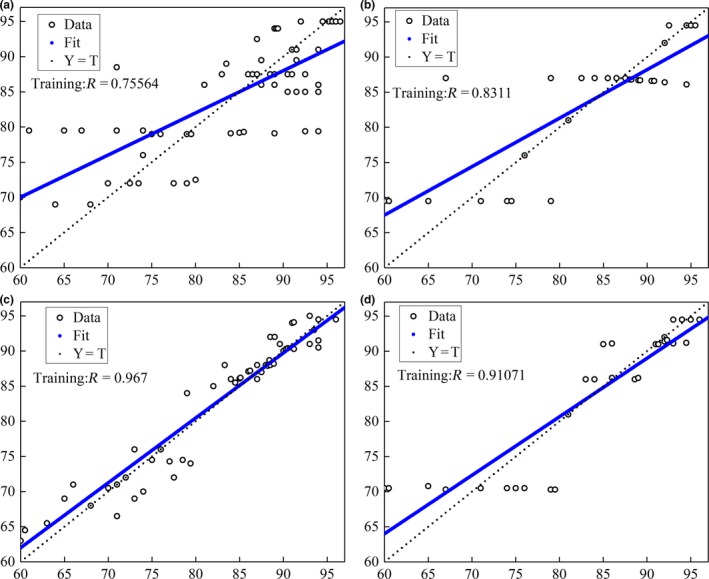
Training and validating results of BPNN with 9 peaks and 4 peaks. (a) BPNN training with 9 peaks. (b) BPNN validating with 9 peaks. (c) BPNN training with 4 peaks. (d) BPNN validating with 4 peaks

In the BPNN model, nine peaks were also used at the beginning for regression to the sensory scores. The results are shown in Figure 4, where a *R* ^2^ of .76 and *SD* of 5.82 were obtained which showed a relatively low correlation between them. To improve the BPNN model, C7, C9, C12, and C13 were again selected to train the BPNN model (Figure 4), where a *R* ^2^ of .97 and *SD* of 2.87 were obtained which showed a higher correlation. When validation was conducted, a *R* ^2^ of .91 and *SD* of 2.85 were achieved.

By comparison of MLR and BPNN regression models, it can be found that in both prediction and validation, BPNN had a higher *R* ^2^ and low *SD*, showed its advantage over MLR. The selection of C7, C9, C12, and C13 could improve the regression models. As a conclusion, BPNN with four peaks of C7, C9, C12, and C13 resulted a good model and can replace the sensory scores in high accuracy.

Through the analysis of 90 different grades' samples, the measurement results of GC‐MS and zNose™ were highly correlated and could replace each other. By comparison of two models, BPNN with four zNose™ peaks of C7, C9, C12, and C13 could replace the sensory scores in high accuracy.

### 3.2 Flavor difference analysis of Fenjiu products of three styles

The flavor difference of Fenjiu products would be investigated. The Fenjiu samples of three styles (A, B, and C) produced in the same year but in different months were included (Table 1). Although zNose™, spirit tasters, and GC‐MS analysis were all conducted in our experiments, only zNose™ data are reported here, as it had been proved to have acceptable correlation with the other two methods.

**Figure 5:**
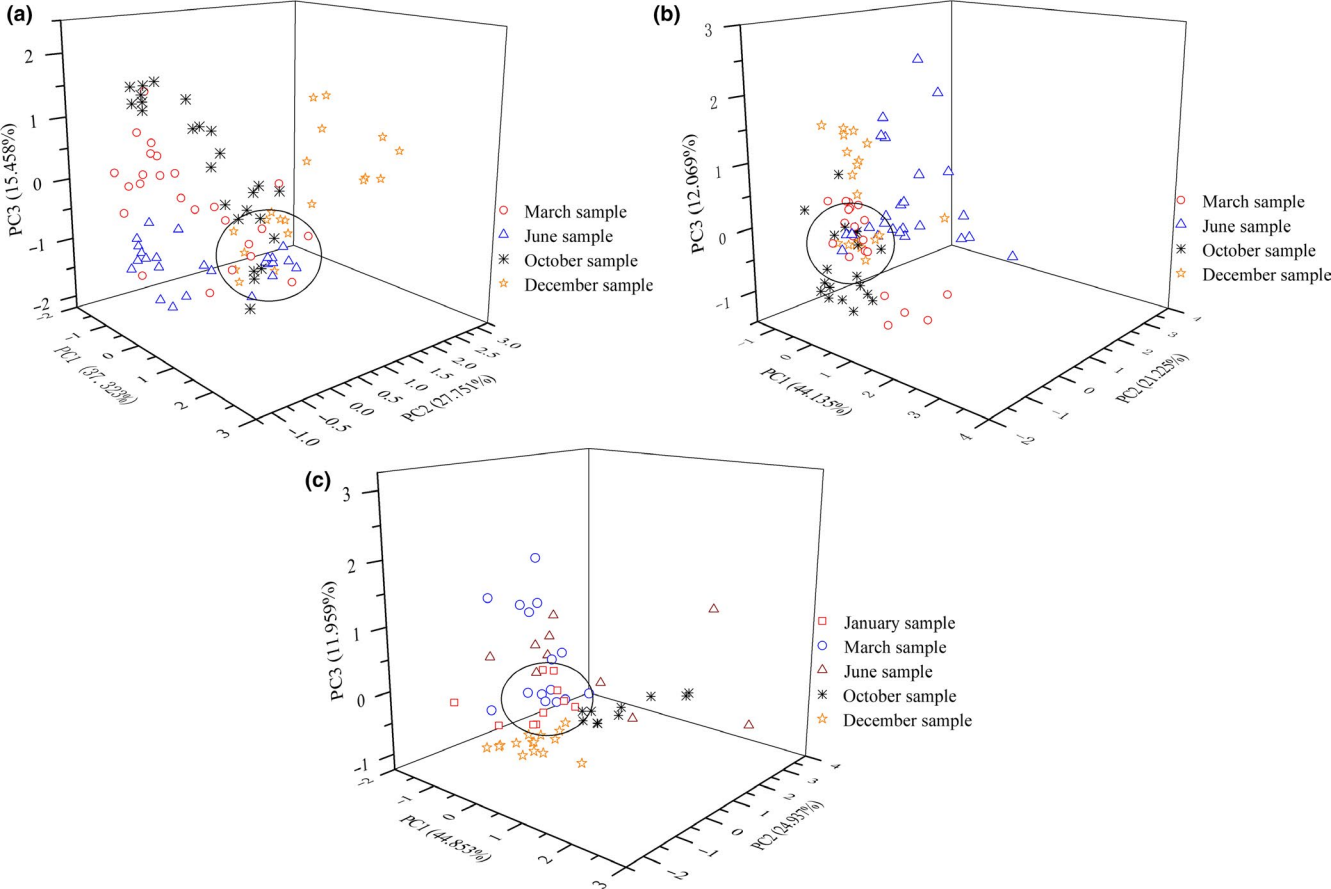
PCA score plots of different style Fenjiu products. (a) A‐style. (b) B‐style. (c) C‐style

The nine peak area values detected by zNose™ from A, B, and C were analyzed with PCA, and variation explanation rates are shown in Table 5. It can be observed that the first three variables can reflect the most difference of the initial nine variables. Score plots were graphed with PC1, PC2, and PC3 as three axes and shown in Figure 5. A sphere center was constructed where the summed distances to the center from each point was the minimum. A spherical surface was further graphed around the sphere center with a fixed distance (Fan et al., 2018). Then, samples were separated into two groups: Those inside the sphere were considered having the similar flavor, hence defined as homogeneous samples; those outside were recognized as heterogeneous samples. The ratio of heterogeneous samples over total samples was defined as dispersion ratio. All the Fenjiu products were conducted the same analysis, and results are shown in Table 6. Among three Fenjiu styles, B has the minimum averaged dispersion ratio of 41.18%, which indicated a small flavor difference. At the same time, the largest difference happened in Fenjiu A, where an averaged dispersion ratio of 55.79% was presented. For each group of A, B, and C, the minimum difference occurred in June, October, and October, respectively, while in March, June, and December their quality has the largest difference. Hence, the uniformity and homogeneity of the same style's Fenjiu products need to be improved for better products.

**Table 5:** Variation explanation of the first three principal components

Products' style	PC1 (%)	PC2 (%)	PC3 (%)	Summed scores (%)
A	37.32	27.75	15.46	80.53
B	44.14	21.22	12.07	77.43
C	44.85	24.94	11.96	81.75

**Table 6:** Dispersion ratio of Fenjiu products

Products' style	Production month	Dispersion ratio (%)	Averaged dispersion ratio (%)
A	3	68	55.79
	6	45	
	10	52	
	12	56	
B	3	42	41.18
	6	56	
	10	40	
	12	50	
C	1	50	48.3
	3	46.7	
	6	50	
	10	40	
	12	53.3	

### 3.3 Flavor difference analysis of base liquors

**Figure 6:**
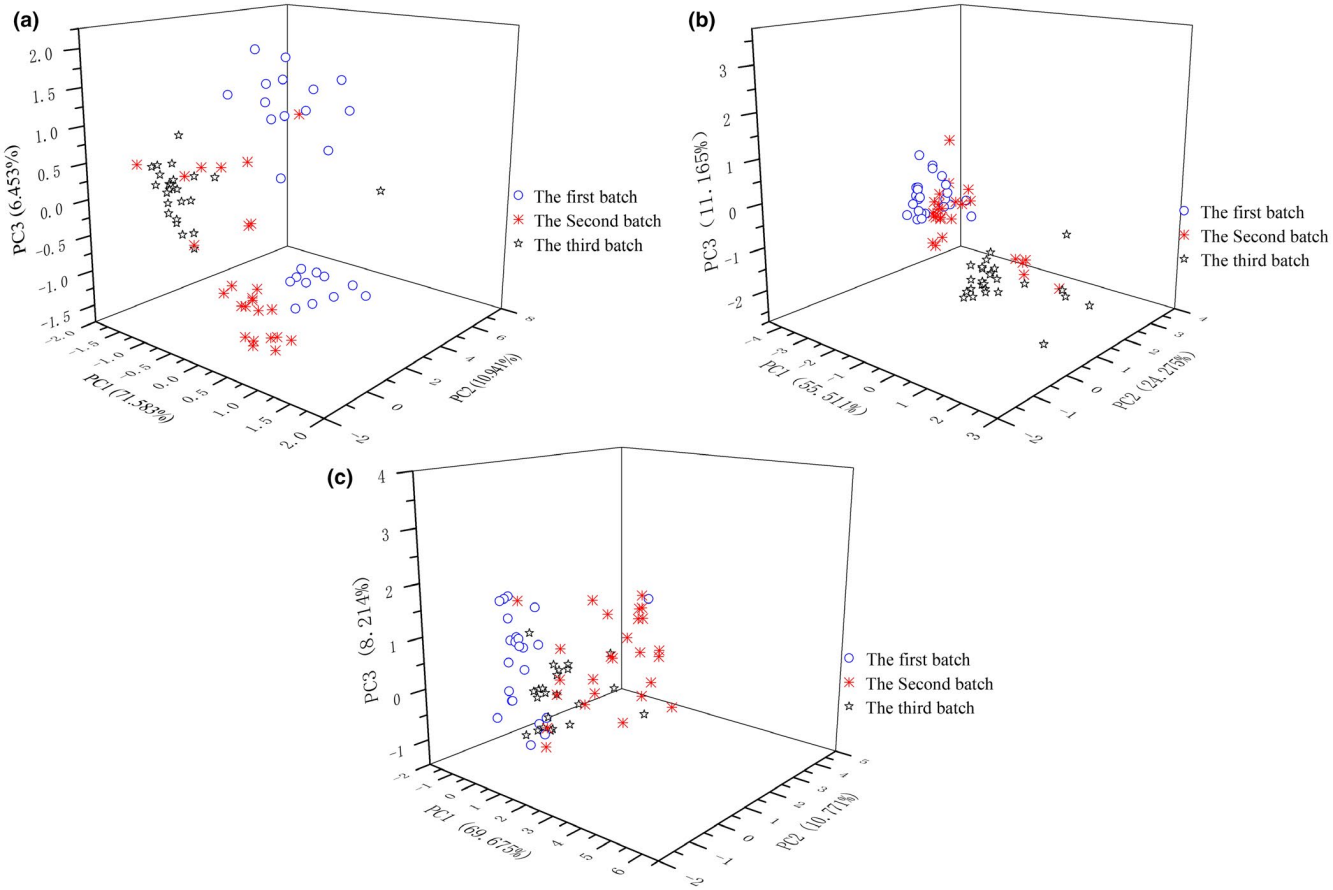
PCA score plots of three base liquors. (a) Dazongjiu. (b) Dajiu. (c) Daijiu

Consistent quality of the product would be more recognized, while the diversity and complexity of raw materials, workers' experience, equipment, and aging condition cause the flavor differences of the base liquors. Three base liquors, called Dazongjiu, Dajiu, and Daijiu (Table 1), were used for blending Fenjiu B‐style products; hence, they were detected with zNose™. Their respective nine peak areas were analyzed with PCA to investigate the difference (Figure 6), as well as ANOM for analysis of MN and CV.

The difference of Dazongjiu samples produced in three batches was evaluated with PCA (Figure 6a). PC1, PC2, and PC3 explained 88.98% difference, and a 3‐dimension graph of them showed that three batches samples majorly distributed in three different areas. PCA of zNose™ data showed that the three batches of the Dazongjiu have obvious difference and overlapping. The area of I included the most samples from the 3rd batch and a few from the 2nd batch; the area of II included the rest 2nd batch and some from the 1st batch; the area of III included the rest of the 1st batch. The largest internal difference occurred in the 1st batch which distributes in two different areas in PCA plot. Most of the 3rd batch was separated into the 1st and the 2nd area.

The difference of 76 Dajiu produced in three batches was also evaluated with PCA. PC1, PC2, and PC3 explained 90.95% difference, and a 3‐dimension graph of them showed that three batches samples majorly distributed in two areas (Figure 6b). The area of I included all samples from the 1st batch and the most from the 2nd batch; the area of II included the rest from the 2nd batch and the most from the 3rd batch. This result showed that the three batches of the Dajiu also have difference among them.

The difference of 76 Daijiu produced in three batches was evaluated with PCA too. The first three variables explained 88.66% difference. A 3‐dimension graph of PC1, PC2, and PC3 showed that three batches samples majorly distributed in two areas near each other (Figure 6c), indicating a small difference and overlapping among them.

Figure 6 showed the difference of three base liquors in three batches. The biggest inconformity acted in Dazongjiu and Dajiu also has difference among different batches, and Daijiu showed relatively uniformity. That difference might be the reason causing the variation of Fenjiu finished products.

Analysis of means showed that by means of MN and *SD*, C7, C9, and C13 had large variance (Table 7). This implies that the three peaks can be used in automatic blending system. If their difference could be minimized, the difference existed in different Fenjiu products would be decreased in a significant degree.

**Table 7:** ANOM of base liquors detected with zNose™

Peak	Batch	Dazongjiu			Dajiu			Daijiu		
		MN	*SD*	CV	MN	*SD*	CV	MN	*SD*	CV
C6	1	496.0	52.7	0.106	588.0	47.2	0.080	499.1	37.6	0.075
	2	575.8	72.5	0.126	689.3	100.2	0.145	592.4	86.8	0.147
	3	976.5	83.7	0.086	983.3	208.9	0.212	939.2	78.7	0.084
C7	1	4,848.8	740.0	0.153	3,585.3	494.2	0.138	3,632.1	400.1	0.110
	2	3,982.9	671.8	0.169	2,709.7	896.1	0.331	2,993.4	585.4	0.196
	3	4,288.8	242.4	0.057	2,148.8	1,053.6	0.490	3,003.6	396.7	0.132
C8	1	1,425.0	152.9	0.107	1,418.4	193.8	0.137	4,436.8	604.0	0.136
	2	1,371.6	125.9	0.092	1,285.0	271.7	0.211	4,482.1	601.1	0.134
	3	1,066.0	87.1	0.082	1,185.6	195.1	0.165	4,265.3	212.3	0.050
C9	1	2,442.1	388.5	0.159	618.1	472.0	0.764	1,498.8	612.3	0.409
	2	1,740.4	495.6	0.285	744.1	355.7	0.478	1,541.4	288.1	0.187
	3	2,616.6	619.0	0.237	1,773.8	635.7	0.358	2,065.7	300.7	0.146
C10	1	701.8	95.1	0.136	921.7	141.5	0.154	857.8	148.4	0.173
	2	641.4	65.7	0.102	1,821.5	374.3	0.205	859.1	179.3	0.209
	3	540.2	56.8	0.105	1,500.7	203.6	0.135	525.4	60.1	0.114
C11	1	1,262.0	201.0	0.159	588.5	83.7	0.142	702.4	254.9	0.363
	2	983.4	220.4	0.224	579.2	115.6	0.200	567.0	222.3	0.392
	3	503.7	153.7	0.305	390.5	90.3	0.231	200.5	48.5	0.242
C12	1	953.6	227.4	0.238	869.1	115.3	0.133	750.9	38.0	0.051
	2	702.0	179.0	0.255	920.6	312.0	0.339	708.8	89.7	0.126
	3	1,254.2	493.3	0.393	1,051.3	369.0	0.351	786.5	58.8	0.074
C13	1	356.2	141.7	0.398	297.0	117.6	0.396	251.8	159.8	0.635
	2	379.8	160.7	0.423	643.2	316.9	0.493	388.6	167.6	0.431
	3	737.1	173.4	0.235	857.6	461.7	0.538	565.5	79.1	0.140
C14	1	241.6	51.4	0.213	291.6	57.6	0.198	323.7	72.7	0.221
	2	438.8	183.9	0.419	445.5	75.9	0.171	346.7	50.0	0.144
	3	496.0	62.2	0.125	700.1	123.4	0.175	549.1	93.3	0.169

### 3.4 Automatic blending

The automatic blending system would be used to improve the uniformity of Fenjiu products. The base liquors of Dazongjiu, Dajiu, and Daijiu, as well as pure water, would be used for blending of a “Target” Fenjiu, which was chosen from Fenjiu B‐style products (Figure 1). Benefitted from the fast detection of zNose™, a feedback control period of the automatic blending system was within 5 min: For 3 base liquors and 1 Target Fenjiu, each spent 1 min for flavor detection and 1 min for zNose™ cleaning. The programs were run in LabVIEW, while BPNN algorithm was conducted in MATLAB, and the results were transferred to LabVIEW for process control.

#### 3.4.1 System operation

In the above Section 3.3, ANOM analysis of Dazongjiu, Dajiu, and Daijiu showed that they had different variances but with the three common, with large variated peaks of C7, C9, and C13. Hence, these three peaks would be used as control parameters in the automatic blending system to improve the product uniformity. Less parameters also simplified the system and speed up the control. The steps were as in Figure 2, and the operation repeated until predefined error was reached. Here, the 5% error was used as it was defined by Fenjiu Group in artificial blending operation. Total 19 blended products were obtained for test.

#### 3.4.2 zNose™ test of blended products

**Figure 7:**
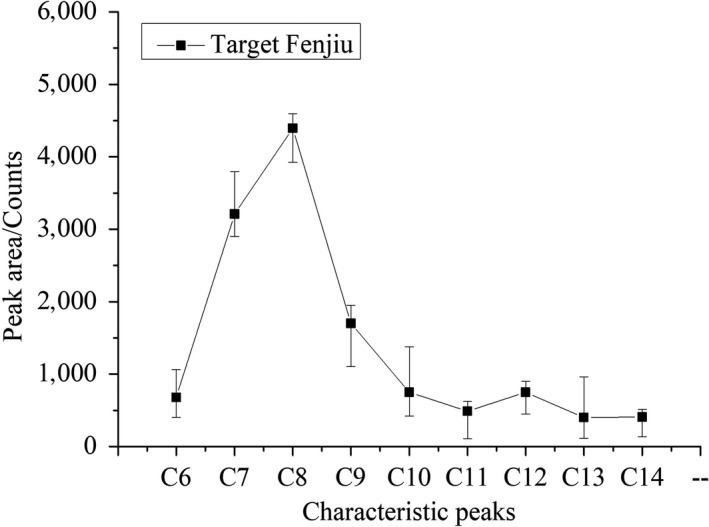
The fluctuation of the zNose™ detected peak areas in blended Fenjiu products

The zNose™ detected nine peak areas of the 19 blended products and shown in Figure 7 with standard deviation. Among them, C7, C9, C10, and C13 had large variations.

**Figure 8:**
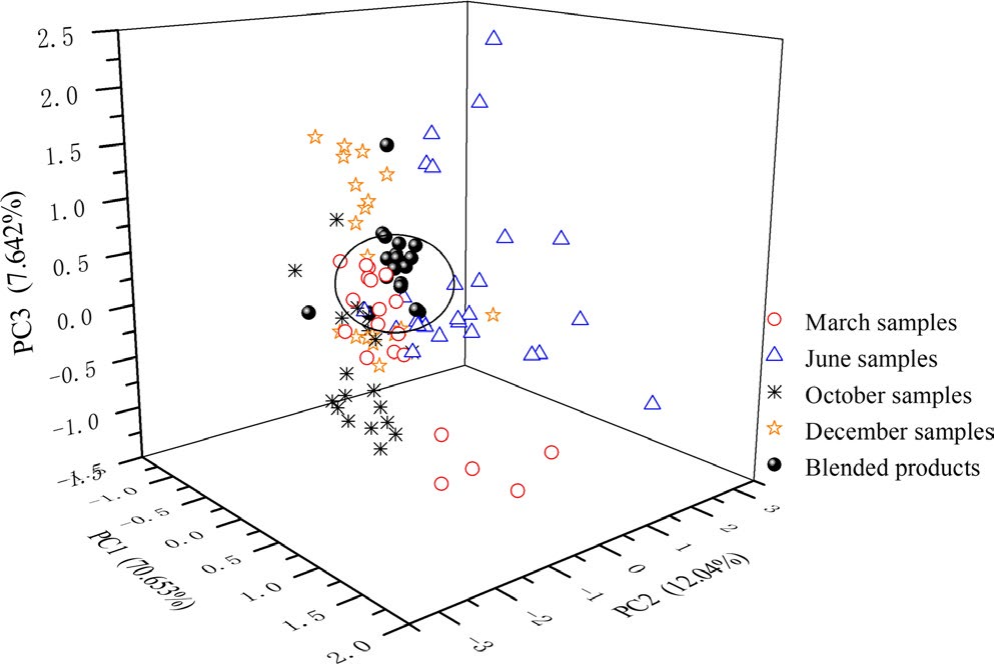
PCA score plots of blended products and Fenjiu B‐style

A PCA was again conducted for the 19 blended products together with 85 Fenjiu B samples. The first three variables explained 90.34% difference and almost covered all the initial information of nine peaks. PC1, PC2, and PC3 contributed to 70.70%, 12.04%, and 7.64%, respectively. Figure 8 showed the three‐dimension score plots of PCA. A sphere surface was graphed whose center was used as the reference point of Fenjiu B. Those inside the sphere were homogeneous samples, and those outside were heterogeneous samples. The results showed that within the 19 blended products, 16 were inside and three were outside. Hence, the dispersion ratio was 15.79%, which was much lower than the 42%, 56%, 40%, and 50% of March, June, October, and December samples. The lower dispersion ratio means less difference to the “Target Fenjiu.” The three heterogeneous points might be the error caused by the blending system.

If not counting the three heterogeneous points and comparing the 16 samples with “Target Fenjiu,” it can be observed that those peaks of C7, C9, and C13 which had large variation previously now have low variation, and the variation is below ±5%.

#### 3.4.3 GC‐MS measurements of the blended products

GC‐MS was also used to check the 16 samples. Total 26 flavor chemicals designated by Fenjiu Group were used for analysis and shown in Table 8. It can be observed that the variation was small for every flavor chemical, which indicated that the automated blending system can improve the blending results in terms of flavor quality.

**Table 8:** Flavor substances' contents

Flavor substance	Target content (mg/L)	Actual blending maximum content (mg/L)	Actual blending minimum content (mg/L)
Acetaldehyde	226.52	236.52	218.72
Acetal	354.80	369.80	344.80
Methanol	90.43	93.93	87.93
Isovaleraldehyde	225.14	233.44	215.84
N‐propanol	132.27	137.47	129.17
Isobutanol	144.13	146.53	139.03
Isoamyl acetate	4.64	4.74	4.44
2‐methyl‐1‐butanol	79.03	81.53	75.93
3‐methyl‐1‐butanol	413.88	427.28	398.68
Isobutyric acid	3.48	3.60	3.33
Butyric acid	1.40	1.43	1.38
Valeric acid	0.87	0.89	0.84
Acid already	3.54	3.66	3.41
Ethyl acetate	48.31	50.41	47.91
Ethyl oleate	17.18	17.58	16.48
Ethyl linoleate	10.42	10.72	10.32
Ethyl caproate	6.38	6.59	6.21
N‐amyl alcohol	7.84	8.04	7.54
Hexanol	4.31	4.45	4.03
Hexyl butyl ester	1.63	1.68	1.61
Ethyl caprylate	4.81	4.93	4.52
Acetic acid	763.60	793.73	734.48
Propionic acid	20.75	21.65	20.04
2,3‐butanediol	26.45	27.16	25.54

#### 3.4.4 Sensory evaluation score of the blended products

Sensory scores were used to evaluate the 16 samples and to compare with 85 finish samples (Table 9). The sensory score of the blended products was 81.5, higher than other products. At the same time, the variance was lower than those of other products. So, the blended products had high flavor evaluation and consistency.

**Table 9:** Sensory scores of Fenjiu finished products and auto‐blended products

Sample	Batch	Average	Variance
B‐style	March	77.5	30.83
	June	73.8	32.77
	October	79.4	10.35
	December	80.6	51.67
Auto‐blended	/	81.5	9.65

## 4 CONCLUSION

In this study, zNose™, GC‐MS, and spirit tasters were used for flavor quality evaluation of different Fenjiu samples. An automatic blending system was designed to improve the uniformity. BPNN was employed for regression and error minimization in blending operation.

For 90 different grade samples, GC‐MS, zNose™, and spirit tasters were all employed to evaluate their flavor quality. zNose™ was found to be correlated with GC‐MS in 95% and hence could be a replacement of GC‐MS in Fenjiu flavor measurement. Two regression models were built between zNose™ and sensory scores from spirit tasters, where BPNN was found to be better with a higher CV of 0.948, indicated that the character variables with BPNN regression model can achieve an acceptable prediction of sensory scores.

For different style products, which were produced in the same year but in different months, zNose™ were used to detect their difference. PCA showed that their dispersion ratio was all more than 40%. Results showed that Fenjiu A‐style, B‐style, and C‐style, although considered as the same products by the producer, have large difference among them.

Three base liquors of Fenjiu B‐style were detected by zNose™. PCA was used for their difference analysis, and ANOM was used for character peaks selection. All three base liquors have variation in their own group. Three peaks of C7, C9, and C13 were found to be the common and large variables by comparison of their MN and CV, and selected as the character peaks for automatic blending.

An automatic blending system was designed for Fenjiu uniformity improvement. zNose™ was employed for fast online flavor detection; C7, C9, and C13 were selected from nine peaks as the character peaks. Three base liquors of Dazongjiu, Dajiu, and Daijiu, as well as pure water, were used to blend a “Target” Fenjiu. Based on BPNN, the “Target” Fenjiu was obtained with an error within 5%. The blended product results were evaluated by zNose™, GC‐MS, and spirit tasters all showed improved of uniformity.

## CONFLICT OF INTEREST

The authors have no conflicts of interest.

## ETHICAL APPROVAL

This study does not involve any human or animal testing.
